# Estimating the Burden of Serious Fungal Infections in Uruguay

**DOI:** 10.3390/jof4010037

**Published:** 2018-03-18

**Authors:** Marina Macedo-Viñas, David W. Denning

**Affiliations:** 1 Sistema Nacional de Investigadores, Agencia Nacional de Investigación e Innovación, Montevideo 11500, Uruguay; 2 National Aspergillosis Centre, Wythenshawe Hospital and The University of Manchester, Manchester M13 9PL, UK; ddenning@manchester.ac.uk; 3 Leading International Fungal Education (LIFE), Cheshire SK10 9AR, UK

**Keywords:** epidemiology of fungal infections, infection burden, Uruguay

## Abstract

We aimed to estimate for the first time the burden of fungal infections in Uruguay. Data on population characteristics and underlying conditions were extracted from the National Statistics Institute, the World Bank, national registries, and published articles. When no data existed, risk populations were used to estimate frequencies extrapolating from the literature. Population structure (inhabitants): total 3,444,006; 73% adults; 35% women younger than 50 years. Size of populations at risk (total cases per year): HIV infected 12,000; acute myeloid leukemia 126; hematopoietic stem cell transplantation 30; solid organ transplants 134; COPD 272,006; asthma in adults 223,431; cystic fibrosis in adults 48; tuberculosis 613; lung cancer 1400. Annual incidence estimations per 100,000: invasive aspergillosis, 22.4; candidemia, 16.4; *Candida* peritonitis, 3.7; *Pneumocystis jirovecii* pneumonia, 1.62; cryptococcosis, 0.75; severe asthma with fungal sensitization, 217; allergic bronchopulmonary aspergillosis, 165; recurrent *Candida* vaginitis, 6323; oral candidiasis, 74.5; and esophageal candidiasis, 25.7. Although some under and overestimations could have been made, we expect that at least 127,525 people suffer from serious fungal infections each year. Sporothrichosis, *histoplasmosis*, paracoccidioidomycosis, and dermatophytosis are known to be frequent but no data are available to make accurate estimations. Given the magnitude of the burden of fungal infections in Uruguay, efforts should be made to improve surveillance, strengthen laboratory diagnosis, and warrant access to first line antifungals.

## 1. Introduction

Uruguay is the second smallest country in South America and considered a high-income country by the World Bank. Climate is template and humid, with average temperatures of 17 °C. The Uruguayan economy is dominated by agriculture and livestock production.

Fungal infections are caused by opportunistic and non-opportunistic fungi, including the primary pathogens *Paracoccidioides brasiliensis* and *Histoplasma capsulatum*, which are endemic in the country. The recognized spectrum of fungal species is not as wide as in other Latin American countries [1], although the real magnitude of these infections is not well known. Notification of fungal infections is not mandatory in Uruguay, so there are no comprehensive registries of such infections. Some of the opportunistic invasive mycosis are probably growing as the immunocompromised population increases, due to the increase in solid organ and bone marrow transplant and the use of immunosuppressive drugs [2]. No recent publications of the current situation of these infections in our country are available.

We aimed to estimate the burden of serious infectious diseases in Uruguay, considering those that cause higher mortality and morbidity in the population.

## 2. Materials and Methods

Population structure was extracted from the World Bank data for 2016 [3] and from the last national census conducted by the National Statistics Institute in 2011 [4].

Incidence of invasive fungal infections, in general, and of candidemia in particular, were obtained from the National Surveillance Network for invasive fungal infections [5]. The network carries out a laboratory based surveillance on a voluntary basis. At the time of the last report (2013) 22% of all the private and public hospitals (13 out of 59) from different country regions participated. These centers covered about 140,000 hospitalizations per year. Participating hospitals send fungal isolates from invasive infections. Then, the Hospital Infections Unit at the Health Ministry collects clinical data from each patient.

For other fungal infections, comprehensive searches of government and scientific societies communications were performed (grey literature), and published literature was searched on Pubmed, Google Scholar, and the Latin American search engines for scientific literature: Scielo and Lilacs. The following terms were used, both in Spanish and English: “fungal infections/invasive fungal infections + Uruguay”, “cryptococcal meningitis/ cryptococcosis/ *Cryptococcus* + Uruguay”, “pneumocystosis/*Pneumocystis* + Uruguay”, “aspergillosis/*Aspergillus* + Uruguay”, “allergic bronchopulmonary aspergillosis/ABPA + Uruguay”, “severe asthma with fungal sensitization/SAFS + Uruguay”, “candidemia/candidiasis/*Candida* invasive infections + Uruguay”, HIV opportunistic infections + Uruguay”, “*histoplasmosis*/*Histoplasma* + Uruguay”, “paracoccidioidomycosis/*Paracoccidioides* + Uruguay”, “sporotrichosis/*Sporothrix schenckii* + Uruguay”, “zygomycosis + Uruguay”, and “tinea capitis/tinea corporis/tinea pedis/dermatophytosis + Uruguay”.

Data on underlying conditions were obtained from national registries on tuberculosis [6], HIV/AIDS [7], hematological malignancies [8], and the national institute of highly specialized medicine (Fondo nacional de recursos (FNR)) which coordinates transplantation procedures in the whole country [9]. When no data existed, risk populations were used to estimate frequencies of fungal infections, using the previously described methodology by Leading International Fungal Education (LIFE) project [10].

## 3. Results and Discussion

The Uruguayan population in 2016 was estimated at 3,444,006 inhabitants [3] of which 73% (2,522,059) were older than 17 years, 40% were aged 40 or older (1,381,046) and 22% (757,681) were children younger than 15 [4]. Women younger than 50 represented 35% of the whole population and 67% of all women [4]. Demographic characteristics and size of populations at particular risk for serious fungal infections are shown in Table 1.

Twelve thousand people were living with HIV in 2016 [7]. Mansilla et al. reported that between 1983 and 1991, 6 of 107 patients (5.6%) attending the University Infectious Diseases Service (SEIC) with AIDS, according to CDC criteria at the time [11], presented with cryptococcal invasive infections. Five of the six patients were already known to be in the AIDS stage of the infection. For the sixth patient, it was the marker disease [12]. This population represented about the half of the HIV diagnosed patients at the time [13], when the Uruguayan population was of 2,955,241 habitants. This means the annual incidence in the 1990s was 0.41 cases of cryptococcosis/100,000 habitants. Later, the same author analyzed a series of 172 patients attending the SEIC between 1986 and 1995. Ten percent of them (17/162) developed cryptococcosis at some point during follow up, and for 3% (5/172) this infection was the first disease indicating AIDS [13]. More recently, a laboratory based study reported 147 cases of cryptococcosis (including 135 form HIV patients, 1 from an immunocompetent person, and 1 from a non-HIV immunocompromised patient) from different country regions over six years, between 2006 and 2012 [14]. Based on this last report, assuming that all the isolates from invasive cryptococcosis were received by the author and each represents a different episode, we can estimate about 25 cases/year, which would represent an annual incidence of 2.1/1000 HIV patients and 0.73/100,000 inhabitants. Nevertheless, given that not all isolates will have been referred, the actual incidence is probably significantly higher. This rate is slightly higher than that estimated in other Latina American countries with similar rates of HIV infected population [15,16]. Considering that not only HIV infected individuals develop cryptococcal infections we conclude that the annual incidence may be underestimated.

Forty-eight cases of *Pneumocystis jirovecii* pneumonia (PJP) were reported among HIV patients in 2016 [7]. In 2015, 157 new cases of HIV were diagnosed at AIDS stage, and 28 of them were diagnosed with PJP (18% of AIDS new cases) [17]. The annual incidence of PJP, considering that affecting only HIV patients, is estimated at 2.3/1000 HIV patients and 1.4/100,000 inhabitants. Bienvenu et al. reported that the rate of non-HIV versus HIV patients presenting PJP at the Lyon teaching hospital increased from 1.7 in 2005 to 5.6 in 2013 with the highest risk among patients with hematological and solid malignancies [18]. This fact, coupled with technical difficulties in straightforward microbiological diagnosis, indicates that the actual incidence of PJP is higher. To date, the microbiological diagnosis in Uruguay relies on microscopic examination, which requires highly experienced staff, a good quality respiratory sample and appropriate processing.

The annual incidence of invasive aspergillosis (IA) was estimated based on risk populations published elsewhere. Given that, in 2016, the National Leukemia Registry recorded 126 cases of acute myeloid leukemia [8] and that 30 patients had allogeneic HSCT [9] and assuming a risk rate of 10% for both groups [19], the annual incidence among patients with major hematological conditions would be 0.82/100,000. Approximately 1400 cases of lung cancer are reported each year in Uruguay [20] (age-standardized incidence 27.4/100,000) and most of them are diagnosed at a late stage [21]. Yan et al. estimated that 2.6% of lung cancer patients will suffer from IA [22] which extrapolates to 36 cases in our country. If the risk for SOT is extrapolated from Pappas et al (US TRANSNET study), 6% of heart, 4% of lung and liver, and 0.5% of kidney transplants would be complicated with IA [23]. This would represent a very low number of patients (2/year). The major contribution to the burden of IA is represented by hospitalized patients with chronic pulmonary obstructive disease (COPD). In 2003, the prevalence of COPD was estimated at 19.7% in Montevideo in those aged 40 and over [24]. Different hospitalization rates have been reported for COPD patients [25,26,27,28]. We assumed the worst case scenario of 20% of COPD patients admitted to the hospital each year, as it is the case in some countries from Africa and Asia [26,27]. It has been estimated that 1.3% of COPD patients admitted to the hospital will develop IA [29]. Based on these estimations 226,985 individuals (or patients) were affected COPD and 707 would have acquired IA. Summing all the risk populations described above, the total annual incidence of IA in Uruguay is estimated at 22.4/100,000 inhabitants. This is the highest incidence described in Latin America [15,16,25,30,31]. We believe this is due in part to the high incidence of COPD, chronic respiratory disease and lung cancer affecting our population compared to other countries in the region. Another reason could be that IA notification is not mandatory in most countries, including Uruguay, but informal notification may be more frequent in this small country.

Other clinical presentations of aspergillosis were estimated including chronic pulmonary aspergillosis. It has been previously assumed that 22% of patients with lung cavities and 2% of those without cavities following pulmonary tuberculosis (PTB) will develop chronic pulmonary aspergillosis (CPA) [32]. Patients with PTB are expected to represent ~25% of the total number of CPA cases annually, given that many other pulmonary conditions predispose to CPA, including COPD [33]. In 2015, the honorary commission for fighting tuberculosis and prevalent diseases (CHLAEP) reported that 613 patients were affected with pulmonary tuberculosis, giving an incidence of 3.7/100,000 among HIV infected and 21/100,000 among non-HIV infected, with a total annual incidence 26.2/100,000 [6]. This means that we should expect that 27 new cases of post-PTB CPA occurred in 2016, representing an annual incidence of 0.78/100,000. Assuming a 15% mortality or surgical resection rate, the post-TB 5 year period prevalence will be 85 cases and a total CPA prevalence of 340 cases (2.5/100,000).

Clinical asthma has been estimated at 9% of the adult population [34]. This extrapolates to 226,985 inhabitants in the country. The number of patients estimated to suffer from cystic fibrosis (CF) was estimated at 220, of which 48 were older than 17 [35]. Denning et al. estimated that 2.5% of the adult asthmatic population and 15% of cystic fibrosis population will suffer from allergic bronchopulmonary aspergillosis (ABPA) [36]. In the absence of better estimations in Uruguay, we adopted these assumptions. Then, the prevalence of ABPA is about 165/100,000. Assuming that severe asthma affects 10% of all asthmatics, and that at least 33% of those have fungal sensitization [37], the prevalence of severe asthma with fungal sensitization (SAFS) in Uruguayan population can be expected to be 217/100,000. There are no Uruguayan fungal sensitization data published. Asthma mortality rate is 2.33/100,000. Eighty deaths occur annually. As most of the deaths are in adults, it is likely that at least 50% had SAFS, because of the strong association between SAFS and hospitalization. Nearly 50% of the deaths might have been avoided, had antifungal therapy been used—an inference that needs substantial research to clarify and validate.

ABPA and SAFS are expected to be unusually high due to the large asthmatic and CF adult populations. Brazil reports a higher asthma but a smaller CF prevalence giving a somewhat higher ABPA rate. We believe that these estimations are reasonable because the risk population is well projected. There may be some duplication between the ABPA patients with severe asthma and SAFS, but the both the proportion of severe asthma and fungal sensitization rates are conservative.

The National Surveillance Network for invasive fungal infections reported that in 2011 there were 0.75–1.64 cases of candidemia/1000 hospital admissions [5]. Using a ratio of admissions/population of 10–20/1 [38,39,40,41,42] the annual incidence of candidemia for 2011 is estimated at 16.4–32.8/100,000, which represents 565–1130 patients. A similar incidence is reported from Brazil [25]. We assumed that *Candida* peritonitis occurs in half as many patients with candidemia in intensive care unit (estimated to be about 30% of all candidemias), as in France [43]. The estimated annual incidence is 3.69/100,000 (127 cases/year).

Oral candidiasis is estimated presuming that 90% of untreated later stage HIV patients will develop this infection, while esophageal candidiasis would affect 20% of untreated HIV and 5% of anti-retroviral treated [44,45]. In 2016, 47% of the 12,000 HIV patients (5640 patients) were not under treatment. According to Matee estimations, 2565 (74.5/100,000) and 885 patients (25.7/100,000) would have developed oral and esophageal candidiasis, respectively. Nevertheless, given that immediate treatment following diagnosis is not still the rule in Uruguay [46], it cannot be assumed that all untreated patients are at an advanced stage of immune compromise, so these figures are probably overestimations. However, similar estimates were made in other Latin American countries [16,25,30,31].

Rates of recurrent *Candida* vulvovaginitis (rVVC) (4 or more episodes per year) are estimated at 5–9% of adult women [47]. In Uruguay, given a female population younger than 50 of 1,209,722 in 2016, we anticipate between 3512 and 6323 cases/100,000 per year of rVVC.

Sporotrichosis was the most frequent deep mycosis in Uruguay in the 1980s when armadillo hunting started to be frequent [48]. *Histoplasma capsulatum* and *Paracoccidioides brasiliensis* infections are known to be endemic in Uruguay, the first all over the country and the second in specific regions along the main rivers [1]. Isolated cases have been published but it is not possible to make approximations on the burden of these diseases. Likewise, rare cases of mycetoma and chromoblastomycois have been seen, but there are no systematic data to estimate prevalence. Finally, dermatophytosis are known to be frequent, but no recent data is available to make estimations [49].

The estimated burden for serious fungal infections is shown in Table 2.

## 4. Conclusions

Overall, 127,968 persons (3716/100,000) in Uruguay are estimated to suffer from serious fungal infections each year. Immediate life-threatening invasive fungal infections (cryptococcosis, PJP, IA, candidemia, *Candida* peritonitis) account for 2103 cases/year (61/100,000).

A country registry would be advisable for better accuracy, but we think this is a fair estimation of the burden of serious fungal diseases in Uruguay based on precise figures about predisposing conditions registered by local institutions/associations and on published estimated risk.

Even though we cannot say which is the most frequent fungal infection in our population, it is clear that *Candida* spp. and *Aspergillus* spp. infections in different risk populations contribute to a great burden of fungal diseases in our country. While candidemia diagnosis relies mostly on culture and is relatively simple, *Aspergillus* diagnosis is particularly challenging for invasive disease especially and is based not only on culture, whose interpretation could be problematic, but also on serologic testing. Molecular techniques for microbiological diagnosis are becoming increasingly available in the country. This will impact on the measure of different fungal infections like cryptococcal meningitis, PJP, candidemia, and aspergillosis, but we should be careful to not overestimate by misinterpreting the results of these very sensitive new methodologies. Improving diagnostic tools and strengthening the reference laboratory would be measures that could contribute to prompt identification and treatment.

Given the magnitude of the burden of fungal infections in Uruguay, efforts should be made to warrant access to first line antifungals, some of which are not available (posaconazole, micafungin, anidulafungin) or are too expensive to be provided to all in need (caspofungin, liposomal amphotericin B, voriconazole).

Regular monitoring of the incidence of fungal infections and of those at risk may improve the accuracy of these estimates in order to generate public policies to reduce their burden.

## Figures and Tables

**Table 1:** Demographic characteristics and size of populations at risk of serious fungal infections in Uruguay.

	n	% of Total Population
Total population	3,444,006	100
Adults (aged 17 or more)	2,522,059	73
Adults aged 40 or more	1,381,046	40
Women younger than 50 years		35
Children (aged 15 or less)	757,681	22
HIV total	12,000	0.35
HIV, not under treatment	5640	0.16
AML	126	0.004
HSCT	30	0.0009
SOT *	134	0.004
Lung cancer	1400	0.04
COPD	272,066	7.9
Asthma	223,431	6.5
CF in adults	48	0.001
Tuberculosis	61	0.002

HIV: human immunodeficiency virus; AML: acute myeloid leukemia; HSCT: hematopoietic stem cell transplantation; SOT: solid organ transplants; COPD: chronic obstructive pulmonary disease; CF: cystic fibrosis. * Kidney, liver, and heart transplantation procedures.

**Table 2:** Estimated burden of fungal infections.

		Number of Cases	Rate/100,000	Accuracy of Estimation
Cryptococcosis		25	0.73	Probably underestimated
*Pneumocystis jirovecii* pneumonia		48	1.4	Probably underestimated
Invasive aspergillosis		773	22.4	Fair
Chronic pulmonary aspergillosis	Annual incidence	27	0.78	Fair
	5 year prevalence	340	2.5	Fair
ABPA		5682	165	Fair
SAFS		7491	217	Fair
Candidemia		1130	32.8	Probably overestimated
*Candida* peritonitis		127	3.69	
Oral candidiasis		2565	74.5	Probably overestimated
Oesophageal candidiasis		885	25.7	Probably overestimated
Recurrent *Candida* vaginitis		108,875	6323	Probably overestimated
In total		127,968	6870	
